# Supplementary material for: Autonomous and dynamic precursor selection for solid-state materials synthesis
Source: Nat Commun. 2023 Oct 31;14:6956. doi: 10.1038/s41467-023-42329-9 (PMC10618174; doi:10.1038/s41467-023-42329-9)
Supplement: Supplementary file 1 — Supplementary Information [file 41467_2023_42329_MOESM1_ESM.pdf]

Supplementary Information

Autonomous and dynamic precursor selection for solid-state materials synthesis

Nathan J. Szymanski[1,2,†], Pragnay Nevatia[1,†], Christopher J. Bartel[3], Yan Zeng[1,*], and Gerbrand Ceder[1,2,*]

[1] Department of Mat. Sci. & Engineering, UC Berkeley, Berkeley, CA 94720, USA
[2] Materials Sciences Division, Lawrence Berkeley National Laboratory, Berkeley, CA 94720, USA
[3] Department of Chem. Eng. & Mat. Sci., University of Minnesota, Minneapolis, MN 55455, USA
[†] Authors contributed equally
[*] Corresponding authors, yanzeng@lbl.gov (Y. Z.) and gceder@berkeley.edu (G. C.)

Supplementary Note 1

D-optimal design was used as an initial benchmark with which to compare the performance of ARROWS[3] on the $YBa_2Cu_3O_{6.5}$ (YBCO) synthesis dataset. This approach is commonly used in the Design of Experiments (DoE), and it is designed specifically to select the combination of experimental parameters that maximize the determinant of the information matrix. For a more detailed explanation of optimal design and the information matrix, we refer the reader to previous work[1]. Here we perform D-optimal design under the assumption that the yield of our target phase (YBCO) is linearly related to the selection of precursors (P_i) and synthesis temperature (T) through some coefficients (c_i) that can be learned:

$$Yield = \sum_{i=0}^{N} c_i P_i + c_{N+1} T$$

Where P_i is represented using a one-hot encoding as outlined in **Supplementary Note 2** and N is equal to the number of available precursors (*e.g.*, 11 precursors for YBCO). All temperatures are normalized such that values between 600 and 900 °C are mapped onto values between 0 and 1. After building the information matrix for this model, the parameters that maximized its determinant were identified by using the CVXPY and CVXOPT packages within Python. The number of experiments proposed by this approach were progressively increased from one set of parameters up to 188 sets of parameters (*i.e.*, all experiments available in the YBCO space). The number of optimal synthesis routes (yielding pure YBCO) contained within each batch of proposed experiments was identified and used to plot the gray curve shown in **Fig. 3** of the main text.

Supplementary Note 2

Bayesian optimization and genetic algorithms were used to evaluate the performance of traditional active learning algorithms when applied to the $YBa_2Cu_3O_{6.5}$ (YBCO) dataset. For each algorithm, precursors were represented using one-hot encoding. Each one-hot vector contained 11 indices such that each index corresponded to a distinct precursor. A few examples are given below:

BaO: [**1**, 0, 0, 0, 0, 0, 0, 0, 0, 0, 0]

Y_2O_3: [0, **1**, 0, 0, 0, 0, 0, 0, 0, 0, 0]

CuO: [0, 0, **1**, 0, 0, 0, 0, 0, 0, 0, 0]

Precursor sets were accordingly represented by summing the one-hot encoded vectors of the compounds that they contained:

BaO, Y_2O_3, CuO: [**1**, **1**, **1**, 0, 0, 0, 0, 0, 0, 0, 0]

For the Bayesian optimization process, the correlation between these inputs and the yield of YBCO was modeled using a random forest regressor. Training was performed on an initial batch of five experiments, after which new precursor sets were iteratively suggested and their outcomes were added to the training set in a serial, one-by-one manner. The selection of these precursor sets was performed using a purely greedy approach whereby the one with the highest predicted yield was chosen at each iteration. Various acquisition functions including Expected Improvement (EI) and Upper Confidence Bound (UCB) were also tested but did not give improved results.

For optimization with a genetic algorithm, we used a population size of 10 experiments each generation. The initial generation was generated by random sampling from all possible precursor sets. Uniform crossover was applied between generations with a probability of 75% and a mutation rate of 25%. We also used an elitism ratio of 10% to keep the best experiment from each generation. The algorithm was halted when all 10 of the most effective synthesis routes (yielding pure YBCO) were identified.

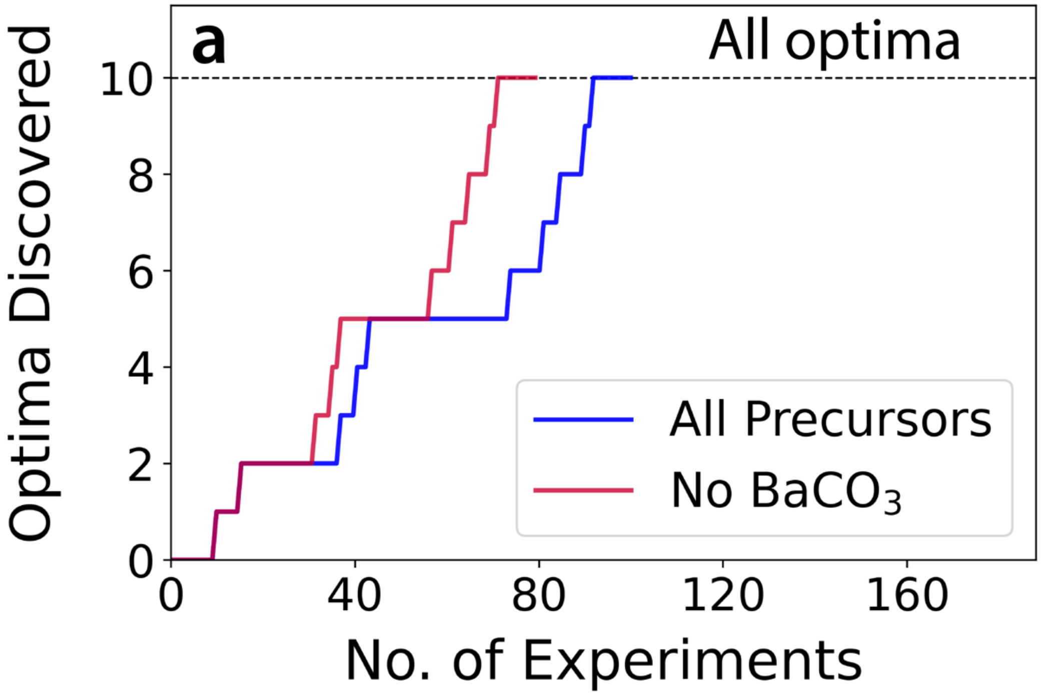

Supplementary Figure 1: Number of optimal synthesis routes for $YBa_2Cu_3O_{6.5}$ (YBCO) that were identified as a function of the experimental iterations required by ARROWS[3]. The blue line represents optimization performed throughout the entire search space, while the red line represents optimization performed in that space while excluding $BaCO_3$ as a precursor. This test is designed to probe the effect of incorporating prior knowledge into the search space, as a domain expert may decide to exclude $BaCO_3$ owing to its high decomposition temperature.

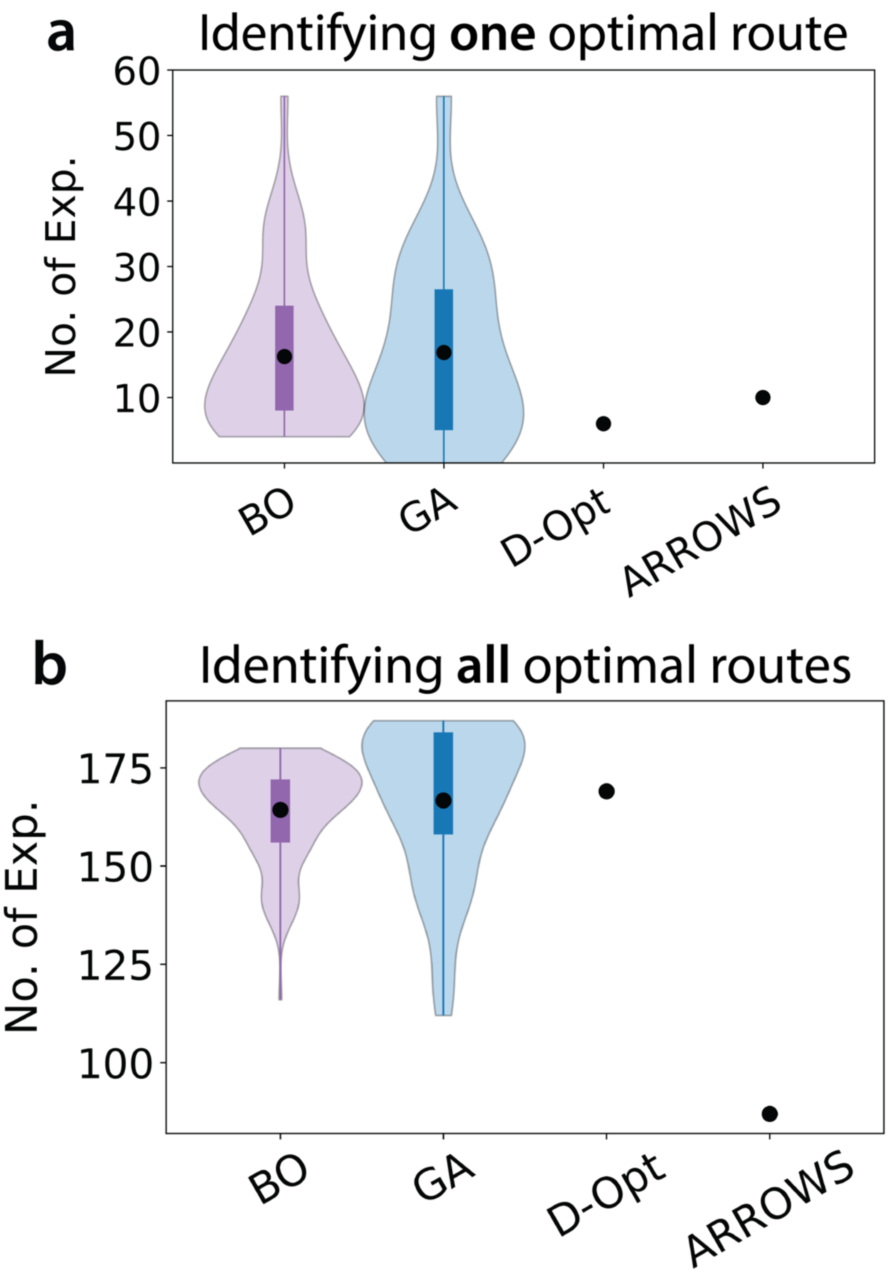

Supplementary Figure 2: Distributions showing the number of experimental iterations required to identify (**a**) at least one optimal synthesis route for $YBa_2Cu_3O_{6.5}$ (YBCO), or (**b**) all ten optimal synthesis routes for YBCO. Results are categorized by the optimization algorithm used to identify these routes. In each violin plot, the embedded box extends from lower to upper quartiles of the distribution. Black dots are used to denote the mean. Because both Bayesian Optimization (BO) and Genetic Algorithms (GAs) are stochastic, the number of iterations required by each method varies substantially depending on the random starting seed. In contrast, D-optimal design (D-Opt) and ARROWS[3] are both deterministic.

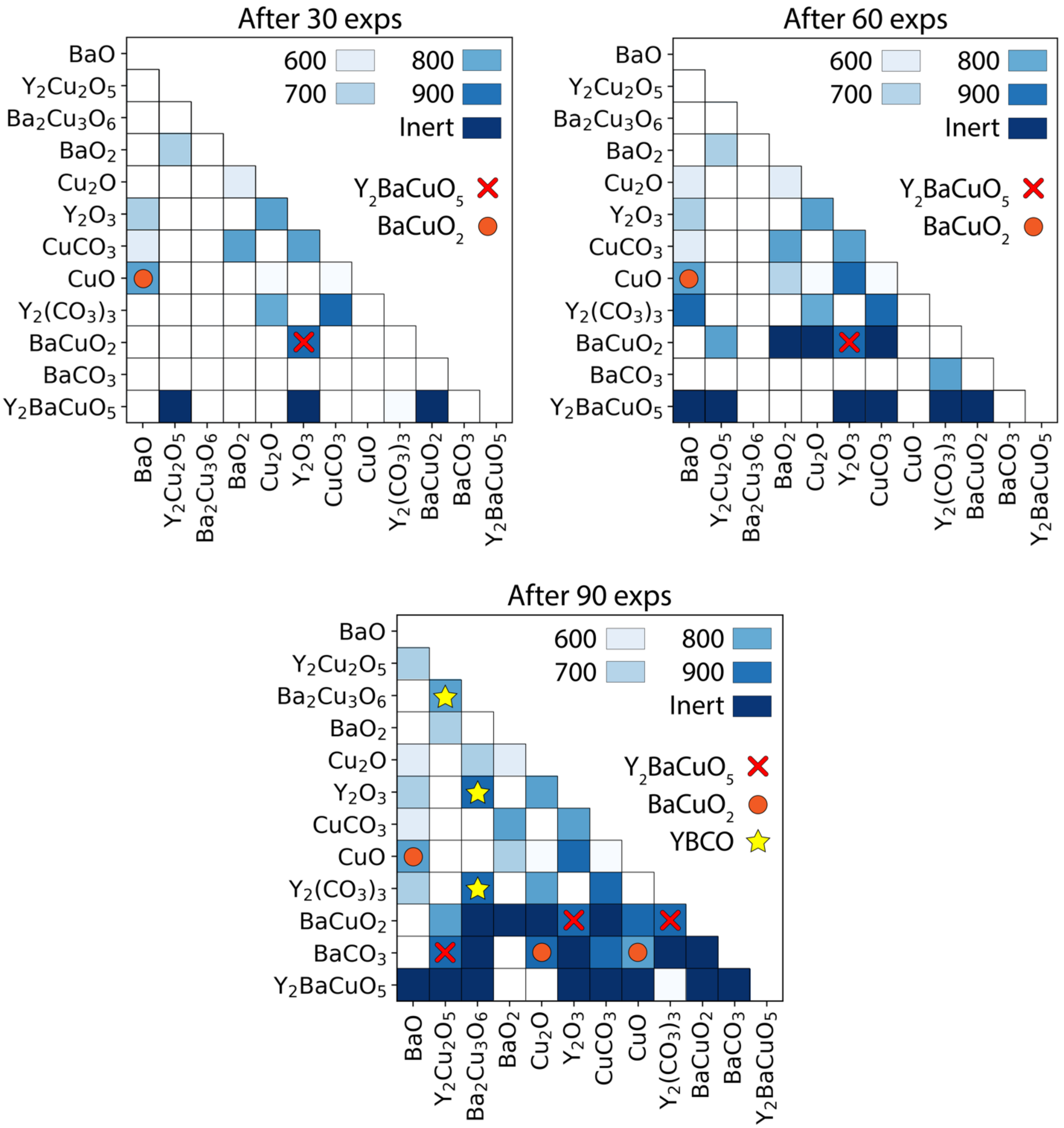

Supplementary Figure 3: Evolution of pairwise reactions identified in the Y-Ba-Cu-O chemical space as more experiments are performed, where each heatmap provides a snapshot of the information learned by ARROWS[3] after it analyzed 30, 60, and 90 experiments. The squares are colored by the temperature (°C) at which a reaction is observed. Inert pairs correspond to phases that do not react within the temperature range considered. Pairs without any data are left blank (white squares). Yellow stars denote pairs that react to produce $YBa_2Cu_3O_{6.5}$ (YBCO). Orange circles and red crosses denote pairs that form impurities, Y_2BaCuO_5 and $BaCuO_2$, respectively.

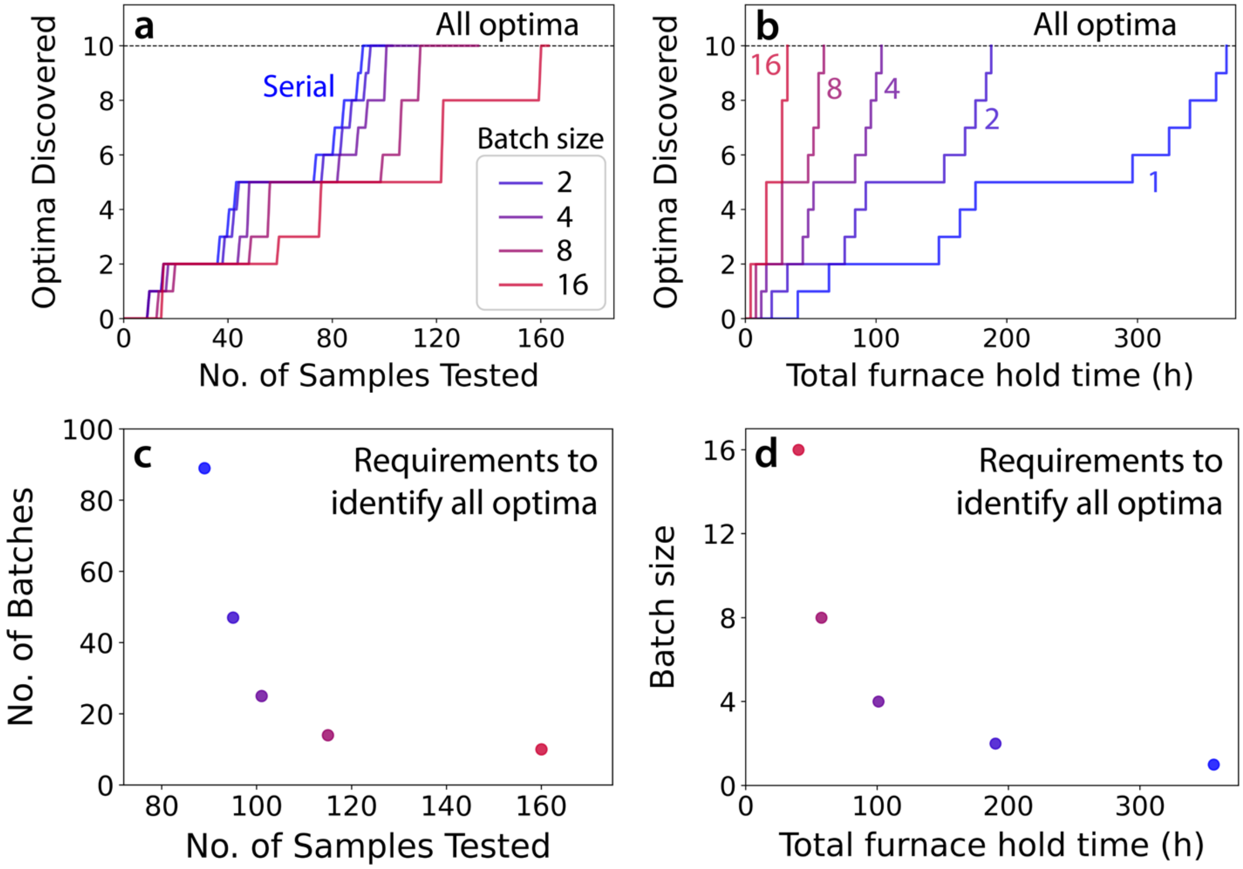

Supplementary Figure 4: Effects of batch size on the optimization of $YBa_2Cu_3O_{6.5}$ (YBCO) synthesis. (**a**) Number of optimal synthesis routes identified with respect to the number of samples queried by ARROWS[3]. Each curve represents an optimization campaign performed with a distinct batch size. (**b**) Number of optimal synthesis routes discovered versus the furnace hold for evaluating the required number of batches. (**c**) Number of batches and samples required to identify all ten optimal synthesis routes, with each dot symbolizing an optimization campaign for a specific batch size. (**d**) Total furnace hold time required to identify all optimal synthesis routes for each batch size.

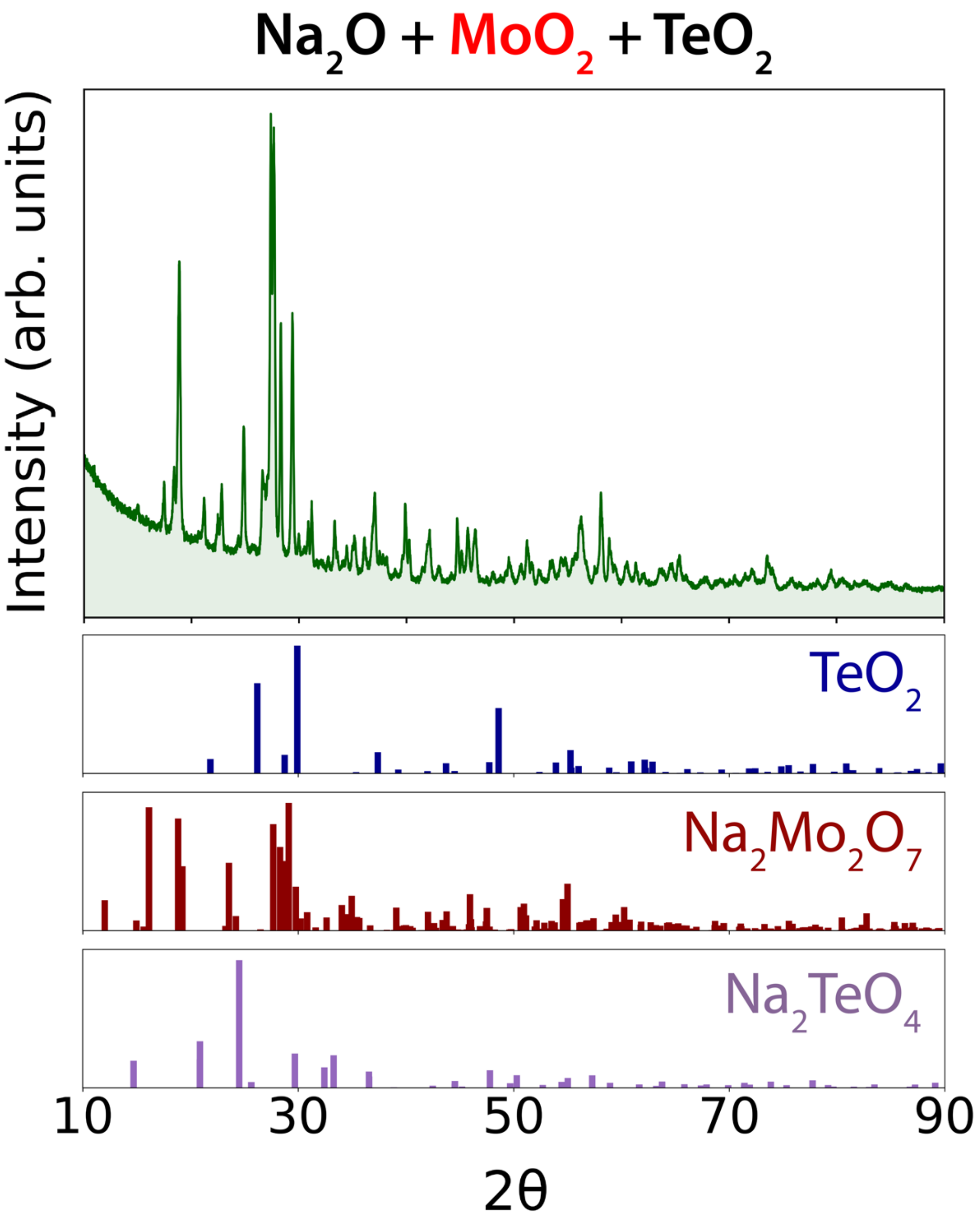

Supplementary Figure 5: X-ray diffraction (XRD) pattern obtained from a synthesis product that was made by heating a precursor mixture of Na_2O, MoO_2, and TeO_2 at 430 °C for 8 h. Reference patterns extracted from the ICSD are also shown for all phases identified in the product. This result serves as a baseline with which to compare the outcome of the optimized precursor set identified by ARROWS[3] – Na_2O, MoO_3, and TeO_2 – which produced $Na_2Te_3Mo_3O_{16}$ (NTMO) without any detectable impurities by following the same synthesis procedure.

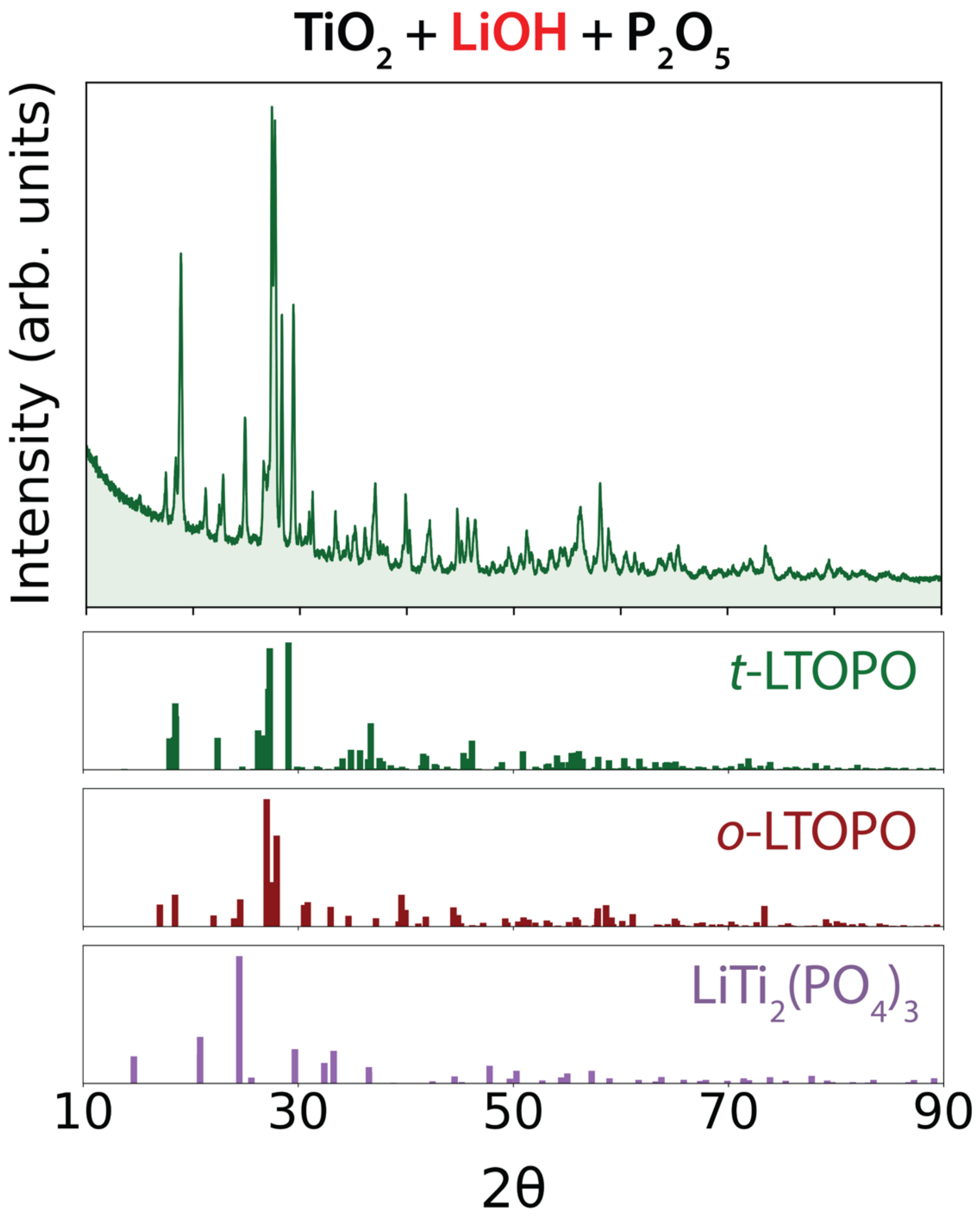

Supplementary Figure 6: X-ray diffraction (XRD) pattern obtained from a synthesis product that was made by heating a ball milled precursor mixture of TiO_2, LiOH, and P_2O_5 at 700 °C for 4 h. Reference patterns extracted from the ICSD are also shown for all phases identified in the product. This result serves as a baseline with which to compare the outcome of the optimized precursor set identified by ARROWS[3] – TiO_2, Li_2O, and P_2O_5 – which produced the desired triclinic polymorph of $LiTiOPO_4$ (*t*-LTOPO) without any detectable impurities while using the same heating profile.

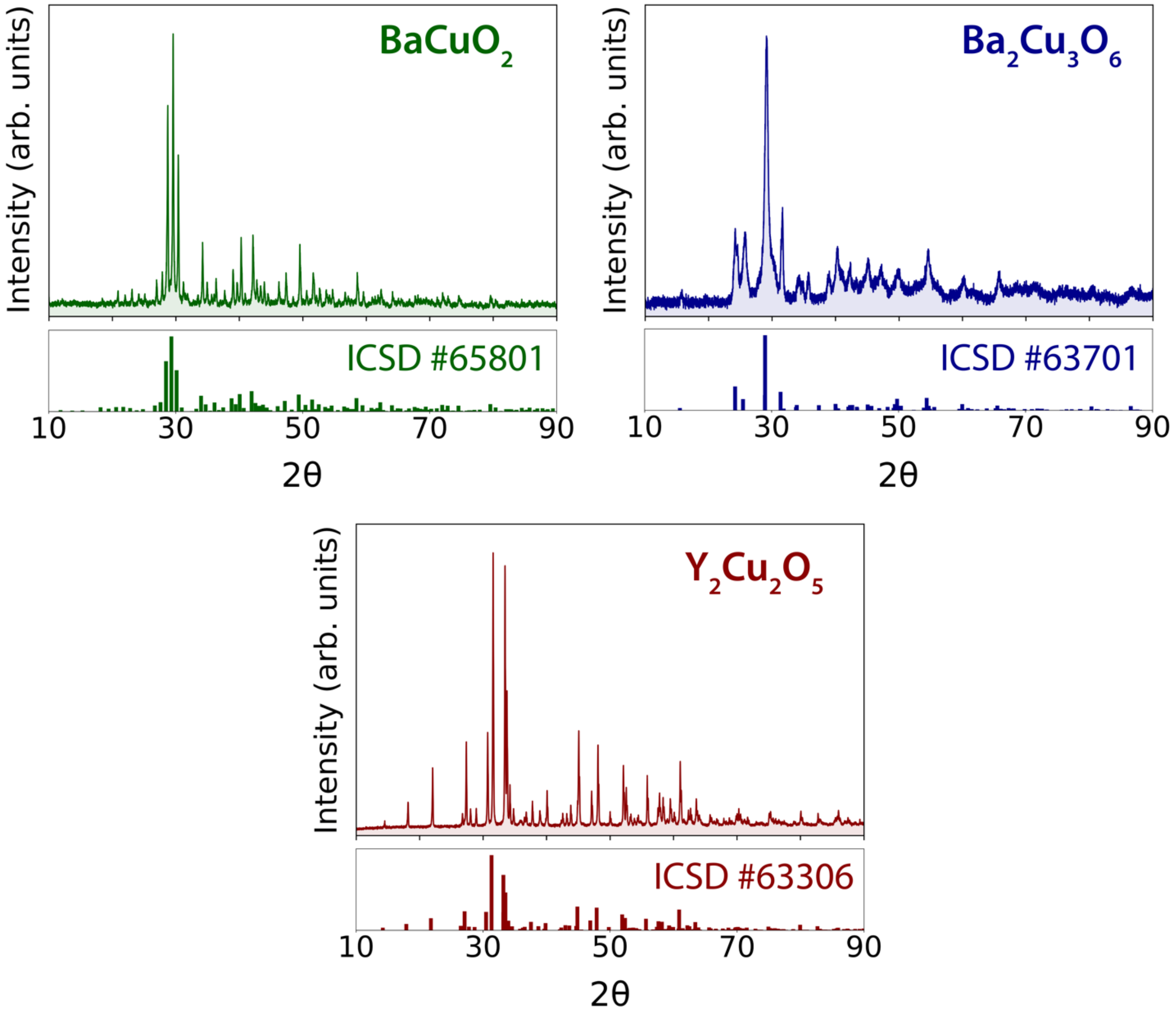

Supplementary Figure 7: X-ray diffraction (XRD) patterns obtained from the synthesized ternary precursors for $YBa_2Cu_3O_{6.5}$ (YBCO). Reference patterns extracted from the ICSD are also shown.

Supplementary Table 1: All precursor sets tested for the synthesis of $YBa_2Cu_3O_{6.5}$ (YBCO). The stoichiometry of each set is determined by the composition of YBCO, in addition to gaseous byproducts that include O_2 and CO_2. Of the 47 precursor sets tested, only 10 resulted in the formation of YBCO without any detectable impurity phases. These 10 sets are shaded green.

Precursor set	Stoichiometry
BaO2, CuCO3, Y2Cu2O5	4, 4, 1
Y2(CO3)3, BaO, CuCO3	1, 4, 6
BaO2, Cu2O, Y2Cu2O5	4, 2, 1
Y2O3, BaO, BaO2, Cu2O	1, 1, 3, 3
BaO2, CuO, Y2Cu2O5	4, 4, 1
Y2(CO3)3, BaO, CuO	1, 4, 6
BaO, Cu2O, Ba2Cu3O6, Y2Cu2O5	12, 4, 4, 5
Y2O3, BaO, Cu2O, Ba2Cu3O6	5, 8, 6, 6
BaO, Ba2Cu3O6, Y2Cu2O5	4, 4, 3
Y2O3, Ba2Cu3O6	1, 2
Y2O3, BaO2, CuCO3	1, 4, 6
Y2O3, BaO, CuCO3	1, 4, 6
Y2(CO3)3, BaO2, CuCO3	1, 4, 6
Y2O3, BaCO3, CuCO3	1, 4, 6
BaO, CuCO3, Y2Cu2O5	4, 4, 1
Y2(CO3)3, BaCO3, CuCO3	1, 4, 6
BaCO3, CuCO3, Y2Cu2O5	4, 4, 1
Y2O3, BaO2, Cu2O	1, 4, 3
Y2O3, CuCO3, BaCuO2	1, 2, 4
Y2O3, BaO2, BaCO3, Cu2O	1, 3, 1, 3
Y2O3, BaO2, CuO	1, 4, 6
BaO2, BaCO3, Cu2O, Y2Cu2O5	2, 2, 2, 1
BaO, BaO2, Cu2O, Y2Cu2O5	2, 2, 2, 1
Y2(CO3)3, CuCO3, BaCuO2	1, 2, 4
Y2(CO3)3, BaO2, Cu2O	1, 4, 3
Y2(CO3)3, BaO2, BaCO3, Cu2O	1, 3, 1, 3

Y2(CO3)3, BaO, BaO2, Cu2O	1, 1, 3, 3
Y2(CO3)3, BaO2, CuO	1, 4, 6
Y2O3, BaCO3, CuO	1, 4, 6
BaCO3, CuO, Y2Cu2O5	4, 4, 1
Y2(CO3)3, BaCO3, CuO	1, 4, 6
Y2O3, BaO, CuO	1, 4, 6
BaO, CuO, Y2Cu2O5	4, 4, 1
Y2(CO3)3, BaO2, Cu2O, BaCuO2	1, 2, 2, 2
BaCO3, Cu2O, Ba2(CuO2)3, Y2Cu2O5	12, 4, 4, 5
Y2O3, BaCO3, Cu2O, Ba2(CuO2)3	5, 8, 6, 6
Y2(CO3)3, BaCO3, Cu2O, Ba2(CuO2)3	5, 8, 6, 6
BaCO3, Ba2(CuO2)3, Y2Cu2O5	4, 4, 3
Y2(CO3)3, BaO, Cu2O, Ba2(CuO2)3	5, 8, 6, 6
Y2O3, CuO, BaCuO2	1, 2, 4
BaCuO2, Y2Cu2O5	4, 1
Y2(CO3)3, CuO, BaCuO2	1, 2, 4
Y2(CO3)3, Cu2O, BaCuO2, Ba2(CuO2)3	3, 2, 8, 2
Y2O3, Cu2O, BaCuO2, Ba2(CuO2)3	3, 2, 8, 2
Y2(CO3)3, Ba2(CuO2)3	1, 2
Y2O3, BaO2, Cu2O, BaCuO2	1, 2, 2, 2
BaO2, Ba2(CuO2)3, Y2Cu2O5	4, 4, 3

Supplementary Table 2: All precursor sets considered for the synthesis of $Na_2Te_3Mo_3O_{16}$ (NTMO). The stoichiometry of each set is determined by the composition of NTMO, in addition to gaseous byproducts that include O_2, CO_2, NH_3, and H_2O. The optimal set identified by ARROWS[3] is shaded green.

Precursors	Stoichiometry
Na2O, TeO2, MoO3	1, 3, 3
Na2O, TeO2, MoO2, O2	2, 6, 6, 3
Na2O2, TeO2, MoO2, O2	1, 3, 3, 1
NaOH, TeO2, MoO2, O2	4, 6, 6, 3
TeO2, MoO2, Na2TeO3, O2	4, 6, 2, 3
Na2CO3, TeO2, MoO2, O2	2, 6, 6, 3
Na2O2, TeO2, MoO2, MoO3	1, 3, 1, 2
Na2O2, TeO2, MoO2, N2H8MoO4	1, 3, 1, 2
Na2O, TeO2, N2H8MoO4	1, 3, 3
NaOH, TeO2, N2H8MoO4	2, 3, 3
Na2O2, TeO2, N2H8MoO4	1, 3, 3
TeO2, MoO2, Na2MoO4, O2	3, 2, 1, 1
NaOH, TeO2, MoO3	2, 3, 3
Na2O2, TeO2, MoO3	1, 3, 3
Na2CO3, TeO2, N2H8MoO4	1, 3, 3
TeO2, Na2TeO3, N2H8MoO4	2, 1, 3
TeO2, Na2MoO4, N2H8MoO4	3, 1, 2
Na2CO3, TeO2, MoO3	1, 3, 3
TeO2, MoO2, Na2Mo2O7, O2	6, 2, 2, 1
TeO2, MoO3, Na2TeO3	2, 3, 1
TeO2, N2H8MoO4, Na2Mo2O7	3, 1, 1
TeO2, MoO3, Na2MoO4	3, 2, 1
TeO2, MoO3, Na2Mo2O7	3, 1, 1

Supplementary Table 3: All precursor sets considered for the synthesis of $LiTiOPO_4$ (LTOPO) in its triclinic polymorph. The stoichiometry of each set is determined by the composition of LTOPO, in addition to gaseous byproducts that include O_2, CO_2, NH_3, and H_2O. The optimal set identified by ARROWS[3] is shaded green.

Precursor set	Stoichiometry
Li2O, TiO2, P2O5	1, 2, 1
TiO2, LiOH, N2H8HPO4	1, 1, 1
Li2O, TiO2, N2H8HPO4	1, 2, 2
Li4Ti5O12, LiOH, N2H8HPO4	1, 1, 5
Li2O, Li4Ti5O12, N2H8HPO4	1, 2, 10
TiO2, Li2CO3, N2H8HPO4	2, 1, 2
Li4Ti5O12, Li2CO3, N2H8HPO4	2, 1, 10
Li2TiO3, Li4Ti5O12, N2H8HPO4	1, 1, 6
Li2TiO3, TiO2, N2H8HPO4	1, 1, 2
TiO2, NH4H2PO4, LiOH	1, 1, 1
Li4Ti5O12, Li3PO4, N2H8HPO4	3, 1, 14
Li2O, TiO2, NH4H2PO4	1, 2, 2
Li4Ti5O12, NH4H2PO4, LiOH	1, 5, 1
TiO2, Li2CO3, NH4H2PO4	2, 1, 2
Li2O, Li4Ti5O12, NH4H2PO4	1, 2, 10
Li4Ti5O12, Li2CO3, NH4H2PO4	2, 1, 10
Li2TiO3, Li4Ti5O12, NH4H2PO4	1, 1, 6
Li2TiO3, TiO2, NH4H2PO4	1, 1, 2
Li4Ti5O12, Li3PO4, NH4H2PO4	3, 1, 14
TiO2, Li3PO4, N2H8HPO4	3, 1, 2
TiO2, Li3PO4, NH4H2PO4	3, 1, 2
TiO2, P2O5, LiOH	2, 1, 2
TiO2, P2O5, Li2CO3	2, 1, 1
Li4Ti5O12, P2O5, LiOH	2, 5, 2
Li4Ti5O12, P2O5, Li2CO3	2, 5, 1

Li_2O, $Li_4Ti_5O_{12}$, P_2O_5	1, 2, 5
Li_2TiO_3, $Li_4Ti_5O_{12}$, P_2O_5	1, 1, 3
Li_2TiO_3, TiO_2, P_2O_5	1, 1, 1
$Li_4Ti_5O_{12}$, P_2O_5, Li_3PO_4	3, 7, 1
TiO_2, P_2O_5, Li_3PO_4	3, 1, 1

References

1. P. F. de Aguiar, B. Bourguignon, M. S. Khots, D. L. Massart, and R. Phan-Than-Luu. D-optimal designs. *Chemom. Intell. Lab. Syst.* **30**, 199–210 (1995).
